# [Retracted] MicroRNA-7 functions as a tumor-suppressor gene by regulating ILF2 in pancreatic carcinoma

**DOI:** 10.3892/ijmm.2025.5542

**Published:** 2025-05-05

**Authors:** Yiliang Bi, Wei Shen, Min Min, Yan Liu

Int J Mol Med 39: 900-906, 2017; DOI: 10.3892/ijmm.2017.2894

Following the publication of this paper, it was drawn to the Editor's attention by a concerned reader that the Transwell cell migration and invasion assay data shown in Fig. 3C on p. 304 were strikingly similar to data appearing in different form in an article written by different authors at different research institutes that had already been published in the journal *Oncotarget*. Upon performing an independent analysis of the data in this paper, the data shown in Fig. 2B were also strikingly similar to data that had appeared in another previously published article. In view of the fact that the abovementioned data had already apparently been published previously, the Editor of *International Journal of Molecular Medicine* has decided that this paper should be retracted from the Journal. The authors were asked for an explanation to account for these concerns, but the Editorial Office did not receive a satisfactory reply. The Editor apologizes to the readership for any inconvenience caused.

